# Ki67 Tumor Expression Predicts Treatment Benefit Achieved by Macroscopic Radical Lung-Preserving Surgery in Pleural Mesothelioma—A Retrospective Multicenter Analysis

**DOI:** 10.3390/cancers16101817

**Published:** 2024-05-10

**Authors:** Sarah Hintermair, Stephanie Iser, Alexander Varga, Melanie Biesinger, Tomas Bohanes, Ali Celik, Muhammet Sayan, Aykut Kankoç, Nalan Akyurek, Betul Öğüt, Elisabeth Stubenberger, Bahil Ghanim

**Affiliations:** 1 Karl Landsteiner University of Health Sciences, Dr. Karl-Dorrek-Straße 30, 3500 Krems, Austria; sarah.hintermair@krems.lknoe.at (S.H.); stephanie.iser@iser.at (S.I.); melanie.biesinger@krems.lknoe.at (M.B.); tomas.bohanes@krems.lknoe.at (T.B.); elisabeth.stubenberger@krems.lknoe.at (E.S.); 2 Department of General and Thoracic Surgery, University Hospital Krems, Karl Landsteiner University of Health Sciences, Mitterweg 10, 3500 Krems, Austria; 3 Department of Pathology, University Hospital Krems, Karl Landsteiner University of Health Sciences, Mitterweg 10, 3500 Krems, Austria; a.varga@ordination-pathologie.at; 4 Department of Thoracic Surgery, School of Medicine, Gazi University, Besevler, Ankara 06500, Turkey; alicelik78@gmail.com (A.C.); drsayann@gmail.com (M.S.); drgangoushev@gmail.com (A.K.); 5 Department of Pathology, School of Medicine, Gazi University, Besevler, Ankara 06500, Turkey; nakyurek2001@yahoo.com (N.A.); betulogut@gazi.edu.tr (B.Ö.)

**Keywords:** pleural mesothelioma, inflammation, Ki67 index, prognostic biomarker, predictive biomarker, multimodality treatment, macroscopic complete resection, cytoreductive surgery, lung-sparing surgery

## Abstract

**Simple Summary:**

Pleural mesothelioma (PM) is characterized by its poor prognosis due to late-stage diagnosis, limited effective treatment options and the lack of biomarkers for treatment decision. The role of macroscopic radical surgery has therefore been questioned. Thus, there is an urgent need for predictive biomarkers aiding clinicians in tailoring more personalized and effective therapeutic strategies. Besides validating the prognostic role of Ki67 expression in tumor samples in 70 PM patients from two different centers with detailed clinical characteristics, here we suggest for the first time Ki67 as a biomarker to predict the patient’s benefit from lung-sparing surgery within multimodality therapy in epithelioid mesothelioma.

**Abstract:**

Pleural mesothelioma (PM), linked to asbestos-induced inflammation, carries a poor prognosis. Therapy ranges from therapy limitation to aggressive multimodality treatment. Given the uncertainty about treatment benefits for patients, this study aimed to assess the role of Ki67 as a prognostic and predictive parameter in PM. Ki67 was measured in the specimens of 70 PM patients (17 female, 53 male) from two centers and correlated to overall survival (OS) and therapy outcome. The median OS was 16.1 months. The level of Ki67 expression was divided into low (≤15%) and high (>15%). A low value of Ki67 expression was associated with a longer OS (Ki67 ≤ 15%: 31.2 (95% CI 6.5–55.8) months vs. Ki67 > 15%: 11.1 (95% CI 7.7–14.6) months, *p* = 0.012). The 5-year survival represents 22% in the low Ki67 expression group, in contrast to 5% in the high Ki67 expression group. We found a significant interaction term of Ki67 with multimodality treatment (*p* = 0.031) translating to an OS of 48.1 months in the low expression Ki67 group compared to 24.3 months in the high Ki67 expression group when receiving surgery within multimodality therapy. Therefore, Ki67 stands out as a validated prognostic and, most importantly, novel predictive biomarker for treatment benefits, particularly regarding surgery within multimodality therapy.

## 1. Introduction

Pleural mesothelioma (PM) is an aggressive neoplasm of the pleura, which is often associated with asbestos exposure [1]. It poses a formidable challenge in the realm of oncology worldwide, characterized by limited treatment options, its poor prognosis and the necessity for reliable prognostic markers [2].

The diagnosis of mesothelioma can be difficult and is often delayed; it can be suspected with CT-imaging but must be confirmed with a biopsy [3,4,5]. Immunohistochemical markers including Wilms’ tumor gene 1 WT-1, cytokeratin CK5/6, calretinin, epithelial membrane antigen (EMA), podoplanin, and mesothelin can confirm the diagnosis [6,7]. Three main histological subtypes can be differentiated: epithelioid, sarcomatoid, and biphasic [8,9]. Epithelioid and biphasic mesothelioma make up approximately 75–95% of mesotheliomas [10].

The median survival (OS) of 8 to 30 months for all histological subtypes only is caused by aggressive biological behavior, therapy resistance and the lack of approved screening methods for early disease detection [10]. The therapy regime of PM extends from therapy limitation to aggressive multimodality treatment, which comprises systemic treatment including immune therapy, surgery and radiation [11,12].

There is not enough evidence of established immunohistochemical prognostic markers to routinely assess the prognosis or treatment response in PM at the moment. The authors had already investigated the C-reactive protein and fibrinogen as potential biomarkers and found that both are prognostic and predictive in PM cases undergoing extrapleural pneumonectomy within multimodality treatment more than 10 years ago [13,14,15]. However, regarding the C-reactive protein, the predictive role was not verified in lung-sparing surgery, as published recently [16]. Furthermore, the proliferation marker Ki67 has emerged as a potential candidate for unraveling the intricate landscape of PM progression and has also been suggested as a prognostic biomarker in epithelioid PM by our group before [17]. Since it is still unclear which patient ultimately benefits from which treatment option [4,18,19], the authors want to evaluate the role of Ki67 tumor expression as a prognostic and predictive parameter in PM in times of modern lung-sparing surgery—especially after the MARS 2 trial questioned the role of surgery in PM in general [20].

Ki67 is a nuclear protein, which can be measured in every phase of cell cycle in proliferating cells [11,21]. As a cellular marker of proliferation, it can be detected within the cell nucleus of proliferating cells, whereas its expression is absent in quiescent cells (G_0_-phase) [11]. It is reported to be prognostic in a number of different malignancies such as non-small cell lung cancer, colon cancer and breast cancer [22,23]. A high Ki67 expression and therefore a high proliferation index is a sign of tumor aggressiveness and tumor growth [11,17]. Bitanihirwe et al. described a prognostic value of Ki67 in treatment-naïve PM patients and in patients after induction therapy [24]. Our group state that patients with a high Ki67 value (>15%) had a significantly (*p* < 0.001) shorter median OS (7.5 months) than those with low Ki67 (19.1 months). In multivariate analyses, Ki67 was an independent prognostic marker in PM (hazard ratio (HR): 2.1, *p* < 0.001) [17]. Interestingly, Ki67 was prognostic exclusively in epithelioid (*p* < 0.001) but not in the non-epithelioid subtype [17,25].

However, Ki67 is still not an established tool to assess prognosis in PM patients and is currently the only pathohistological marker of widely accepted prognostic importance is still the histological subtype. Therefore, this study endeavors to validate Ki67 as a prognostic biomarker. In addition, we wanted to challenge the role of Ki67 as a predictive biomarker for the benefit of lung-sparing surgery within multimodality therapy for the first time. We aim to shed light on its capacity to support our understanding of the disease’s clinical behavior and enable personalized therapeutic strategies, including the recently questioned multimodal treatment approach.

## 2. Materials and Methods

### 2.1. Patients

In this retrospective multicenter study, data from 70 patients with PM diagnosis were collected at the Department of General and Thoracic Surgery of the University Hospital of Krems, Austria (38 patients, 54.3%) and the Department of Thoracic Surgery of the Gazi University of Ankara, Turkey (32 patients, 45.7%). The observation period of our study was from 2008 until 2021. The data collection was conducted according to the ethical principles of the Declaration of Helsinki. The patient’s data were analyzed confidentially and pseudonymously. The Commission for Scientific Integrity and Ethics of Karl Landsteiner Private University approved the study under the number 1047/2021 on the 3rd September 2021 and waived the informed consent due to the retrospective study design.

The eligibility criteria for inclusion into the study cohort comprised a histologically proven diagnosis of PM regardless of the histological subtype (epithelioid, biphasic or sarcomatoid), a medical record and documentation that included the treatments received at the participating hospital, and a standard laboratory and clinical parameters as well as Ki67 tumor expression data.

### 2.2. Variables

In this study, we focused on the Ki67 index, which was collected in all 70 patients.

We divided the study cohort into low (≤15%) and high (>15%) Ki67 expression groups according to our previous study by Ghanim et al. and as commonly performed in former studies for better comparison of the data with international publications [8,13,14]. We have previously explored the cut-off analyses and found 15% to be the most accurate to estimate outcome of PM patients. Thus, here we also utilized the previously published cut-off for validation.

The Ki67 expression was assessed and documented ©n surgical specimens and non-surgical specimens like cytological examination in all 70 PM patients to provide a meaningful comparison between all 70 PM patients in both centers. In 46 patients (65.7%) the Ki67 value was analyzed before any treatment like induction, while in 14 patients (20.0%), the Ki67 expression was evaluated post-treatment in the multimodality setting. A total of 10 patients (14.3%) did not receive multimodality treatment, and here the Ki67 expression was analyzed only on the surgical specimen.

The used antibody was “Anti-Ki-67 (30–9) Rabbit Monoclonal Primary Antibody” by the company Roche (platform BenchMark Ultra).

Clinical characteristics (gender, age, histology, stage, treatment) were correlated to overall survival (OS). Survival of the study cohort was subgrouped regarding Ki67 and therapy modalities. We divided therapy options into multimodality treatment and other therapy options, which included chemotherapy, immunotherapy, radiotherapy, and best supportive care. Multimodal treatment was characterized in both centers as neoadjuvant or adjuvant chemotherapy with cisplatin/pemetrexed, cytoreductive surgery (pleurectomy/decortication or tumor debulking) and additive radiotherapy. We also treated patients with extended pleurectomy/decortication (eP/D) and included them in the group of patients treated with P/D to achieve sufficient numbers in the surgical subgroup for statistical analyses. Therefore, we did not split up patients into eP/D vs. P/D.

Most PM patients received a standard and systemic treatment with pemetrexed plus cisplatin in a multimodal model according to Vogelzang et al. [26].

### 2.3. Statistical Analyses

Statistical analyses were realized with SPSS. *p*-values below 0.05 were considered significant. Overall survival (OS) was defined as time from the date of diagnosis until the time of death or last follow-up and was calculated with Kaplan–Meier analyses. The resulting curves were compared with a log-rank test. Uni- and multivariate Cox regression were applied to identify independent prognosticators and predictors of OS and calculate interaction terms. Univariate survival analyses were performed with categorical and metric data for comparison.

## 3. Results

### 3.1. Ki67 Index

**Figure 1:**
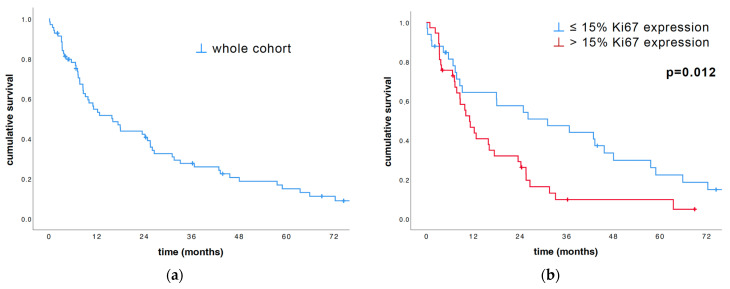
Univariate survival analysis of the entire cohort (**a**), as well as subdivisions based on Ki67 percentage (**b**), was conducted using the log-rank test and illustrated with Kaplan–Meier plots. (**b**) Subdivision of the cohort into low and high Ki67 expression groups states that patients with Ki67 ≤ 15% survived significantly longer than the other patients (*p* = 0.012).

We divided the study cohort into low (≤15%) and high (>15%) Ki67 expression groups according to our previous study [17]. A total of 33 patients (47.1%) were diagnosed with a low-Ki67-expressing tumor and 37 patients (52.9%) with a high-Ki67-expressing one. The total cohort median OS was 16.1 months (95% CI 9.4–22.9, range 0.2–124 months; Figure 1a). The results of univariate survival analyses, analyzed via log-rank as well as Cox regression, proved the prognostic power of Ki67. The Ki67 tumor expression, dichotomized by 15%, yielded significant survival advantages within the group with low Ki67 tumor expression (31.2 vs. 11.1 months, *p* = 0.012; Figure 1b).

### 3.2. Descriptive Statistics and the Ki67 Index

The cohort consisted of 53 male (75.7%) and 17 female (24.3%) patients with histologically verified pleural mesothelioma diagnosis (*n* = 70, median age 66 years, range 42–88 years). A total of 26 (49.1%) of the male patients were diagnosed with a low Ki67 expression, while 27 male patients (50.9%) had a high Ki67 expression when they were diagnosed. According to the median age, we divided our cohort into two groups (age ≤ 66 years and age > 66 years).

We also divided our patients according to histological subtype. A total of 60 patients (85.7%) were diagnosed with an epithelioid subtype, and only 10 patients (14.3%) with a non-epithelioid subtype (comprising mixed and sarcomatoid subtype). Out of the 60 patients with epithelioid subtype, half (30 patients) had a low Ki67 expression. Within the non-epithelioid subtype group, only three patients were diagnosed with low Ki67 expression and seven with high Ki67 expression.

A total of 43 patients (62.3%) were diagnosed at an early stage (I, II), whereas 26 patients (37.7%) received their diagnoses at a late stage (III, IV). The data on one patient’s stage are missing. Of the early-stage patients, 22 (51.2%) had a low Ki67 expression, and 21 (48.8%) had a high Ki67 expression. A total of 10 of the late-stage patients (38.5%) had a low Ki67 expression at the late stage, and 16 patients (61.5%) had a high Ki67 expression.

There was no significant difference in the distribution of the Ki67 index with regard to sex, age, histological subtype or stage.

All these descriptive data are demonstrated in Table 1.

### 3.3. Therapy Modalities and the Ki67 Index

A total of 43 patients underwent cytoreductive surgery in a multimodal setting (61.4%) (Table 1 and Table 2) and another 4 patients (5.7%) received sole surgery. A further 16 patients (22.9%) were treated with best supportive care, and 7 patients (10%) either received systemic chemotherapy, radiotherapy or immunotherapy. Out of the 47 patients who were treated with surgery, 14 patients (29.8%) underwent tumor debulking, while 33 patients (70.2%) were operated with a macroscopic radical pleurectomy/decortication (=P/D). We also treated patients with extended pleurectomy/decortication (eP/D) and included them in the group of patients treated with P/D. Sole surgery is not considered standard therapy in the treatment of patients with PM. However, these four patients were included to gather a representative sample. These four patients received sole surgery due to the fact that two patients passed away within one month after surgery, and two patients were in a bad general condition and had severe comorbidities. So a multimodality concept was not possible or even contraindicated in these four patients.

The patients treated with multimodality therapy (43 patients) received the following therapies in addition to cytoreductive surgery: All 43 patients (100%) received chemotherapy. Out of these, 21 patients (48.8%) had additive radiotherapy and 8 patients (18.6%) were treated additionally with immunotherapy. Multimodal treatment was characterized in both centers as neoadjuvant or adjuvant chemotherapy with cisplatin/pemetrexed, cytoreductive surgery (pleurectomy/decortication or tumor debulking) and additive radiotherapy.

A total of 24 patients (55.8%) treated within the multimodality concept had a low Ki67 expression, while 19 patients (44.2%) had a high Ki67 expression. Furthermore, in the patient group not treated with multimodality therapy (*n* = 27), only 9 patients (33.3%) had a low Ki67 expression, but 18 patients (66.7%) had a high Ki67 expression, which shows that more patients with a low expression were treated in the multimodality concept compared to patients with a high Ki67 expression.

### 3.4. Descriptive Statistics and the Median Overall Survival

The total cohort median OS was 16.1 months (95% CI 9.4–22.9, range 0.2–124 months; Figure 1a). The results of univariate survival analysis, both via log-rank and Cox regression, proved the prognostic power of age and stage at diagnosis.

**Figure 2:**
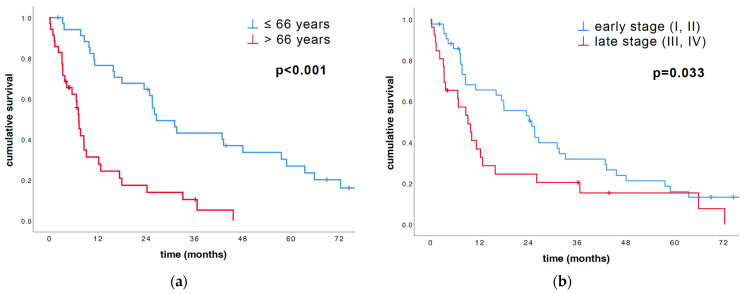
Univariate survival analysis of the subdivision based on age, as well as subdivision of early (I, II) and late stage (III, IV), was conducted using the log-rank test and illustrated with Kaplan–Meier plots. (**a**) Kaplan–Meier survival curve depended on age of the cohort group states that patients younger than 66 years survived significantly longer than patients older than 66 years (*p* < 0.001). (**b**) Kaplan–Meier survival curve divided in early (I, II) and late stage (III, IV) states that patients diagnosed at an early stage (I, II) survived significantly longer than patients at a late stage (III, IV) (*p* = 0.033).

The age, dichotomized by 66 years, yielded significant survival advantages within the younger patients (≤66 years) (26.6 vs. 7.3 months, *p* < 0.001; Table 2 and Figure 2a). As expected, similar results were found at the stage of diagnosis: patients diagnosed at an early stage (I, II) survived significantly longer than patients diagnosed at a late stage (III, IV) (24.9 vs. 9.2 months, *p* = 0.033; Table 2 and Figure 2b). Sex did not have a significant impact on survival for this cohort (*p* = 0.643).

**Figure 3:**
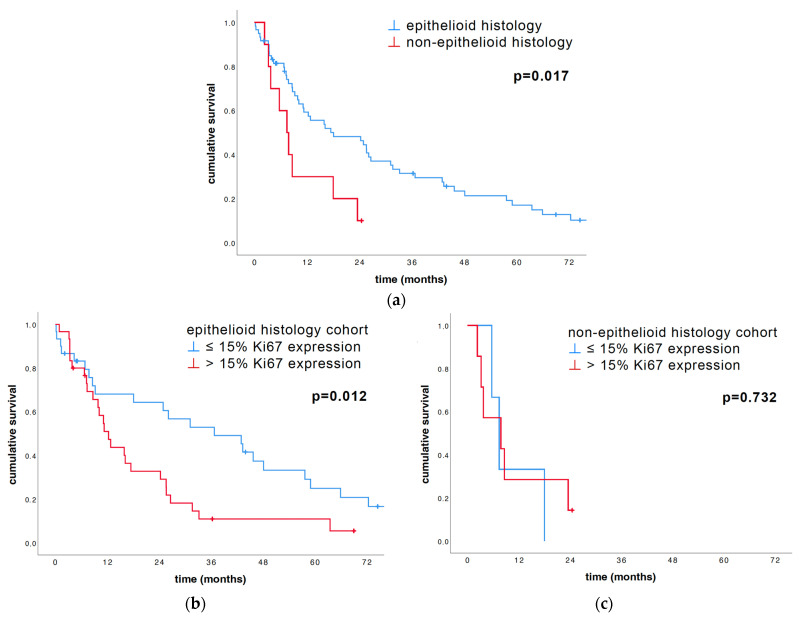
Univariate survival analysis of the histological subtypes’ influence on median OS was conducted with log-rank and illustrated with Kaplan–Meier graphs. (**a**) The subdivision of the cohort into epithelioid and non-epithelioid revealed the strong prognostic impact histology has on survival (18.1 vs. 7.4 months, *p* = 0.017). (**b**) Within the epithelioid subgroup, low Ki67 expression correlated significantly with better median OS compared to high Ki67 expression (36.8 vs. 12.3 months, *p* = 0.012), whereas within the non-epithelioid subgroup, (**c**) Ki67 expression had no significant influence on survival (7.4 vs. 7.8 months, *p* = 0.732).

Again, as anticipated, the histology subtype could prove its significant impact on survival in patients with PM. The epithelioid subgroup survived significantly longer compared to the non-epithelioid subgroup (18.1 months vs. 7.4 months, *p* = 0.017; Figure 3a and Table 2). When looking at the epithelioid subgroup, a low Ki67 expression correlated with a longer median OS (36.8 vs. 12.3 months, *p* = 0.012; Table 2 and Figure 3b). In the non-epithelioid subgroup, a low or high Ki67 tumor expression showed no significant influence on the median OS (7.4 vs. 7.8 months, *p* = 0.732; Table 2 and Figure 3c), which was consistent with previously published data [17].

**Figure 4:**
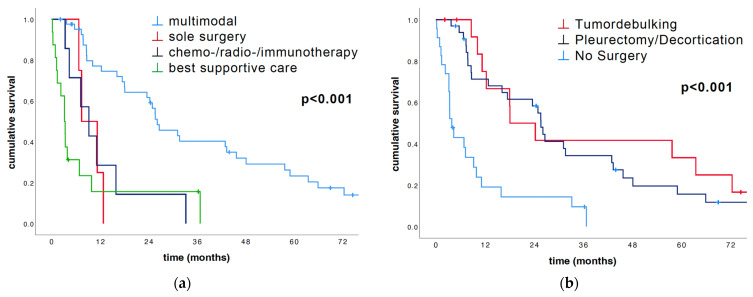
Univariate survival analysis of treatment strategies calculated via log-rank and illustrated with Kaplan–Meier plots. (**a**) The subdivision of the cohort treatment strategies revealed a strong prognostic impact of therapeutic concept on median OS (multimodal 26.1 months vs. sole surgery 7.4 months vs. chemo/radio/immunotherapy 9.2 months vs. best supportive care 3.2 months, *p* < 0.001; Table 2). (**b**) The extent of surgical therapy (tumor debulking and P/D) yielded a significant survival advantage in contrast to no surgery (median OS: P/D 25.6 months vs. tumor debulking 18.1 months vs. no surgery 3.8 months, *p* < 0.001; Table 2).

The different therapy options had a significant impact on OS. Patients treated with multimodality therapy survived significantly longer than patients treated with a sole treatment option (median OS: 26.1 months vs. 6.7 months, *p* < 0.001; Table 2). Looking at the different therapy options, there was a significant difference in the median OS (multimodal: 26.1 months vs. sole surgery: 7.4 months vs. C/R/I: 9.2 months vs. BSC: 3.2 months, *p* < 0.001; Figure 4a and Table 2). The differing surgical options yielded a survival advantage for P/D ahead of tumor debulking and no surgery (25.6 vs. 18.1 vs. 3.8 months, *p* < 0.001; Figure 4b and Table 2).

### 3.5. Multivariate Survival Analysis

To examine the independence of prognostic value of Ki67 expression, a multivariate model adjusted for age, sex, stage, multimodal treatment vs. others, and Ki67 expression was calculated (Table 3). Since Ki67 showed no prognostic value for patients with the non-epithelioid subtype, these patients, and also the patient with missing data regarding histology, were excluded. Therefore, in the following calculation, only 60 patients with the histologically verified epithelioid subtype were included.

Within the multivariate model, therapy options (dichotomized by multimodality treatment) proved to independently influence the risk of earlier death (HR 0.31, 95% CI 0.12–0.82, *p* = 0.019). Sex, stage and Ki67 expression showed no statistically significant relevance after multivariate analyses. This model only includes epithelioid mesothelioma patients.

### 3.6. Interaction between Multimodal vs. Other Therapy and Ki67 Expression

**Figure 5:**
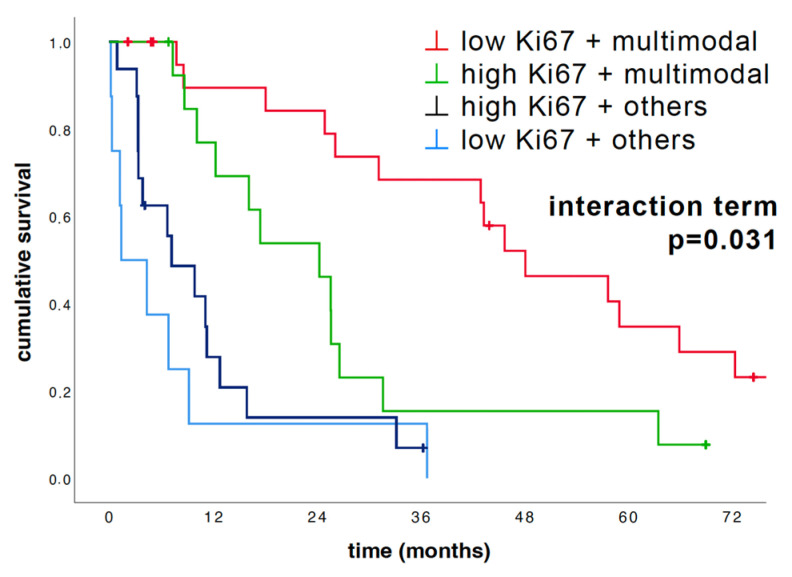
Impact of Ki67 expression on outcome after multimodality therapy and other therapy options. Kaplan–Meier graph illustrating the cohort of epithelioid cases (*n* = 60) divided by Ki67 (cut-off 15%) and multimodal vs. other treatments. The interaction term was determined through multivariate Cox regression, and *p*-values were calculated pairwise using the log-rank test. Median OS differed strongly: low Ki67/multimodal: 48.1 months vs. high Ki67/multimodal: 24.3 months vs. high Ki67/others: 7.2 months vs. low Ki67/others: 1.4 months. Notably, the calculated interaction term was significant (*p* = 0.031).

After failing to show that Ki67 is an independent prognostic marker in our epithelioid cohort, we tested the above mentioned multivariate Cox regression model for interactions between therapy and Ki67. Importantly, we found a significant interaction term between the variables Ki67 expression and multimodal therapy vs. others, indicating that Ki67 is a predictive biomarker. Thus, we tested Ki67 through additional stratification by multimodal therapy vs. other therapy options as illustrated in Figure 5. The survival curves, including the interaction term, indicate a strong interaction between Ki67 expression and outcome of multimodality treatment including lung-sparing surgery. This interaction translated to an excellent OS of 48.1 months in the low Ki67 expression group when receiving multimodality therapy compared to 24.3 months only in the high Ki67 expression group when receiving the same treatment regimen.

The survival of patients in the non-multimodal treatment group was poor in both the high (7.2 months) and the low (1.4 months) Ki67 expression groups.

## 4. Discussion

In the present study, we analyzed the prognostic power of Ki67 tumor expression in patients with pleural mesothelioma. Besides the known prognostic factors, such as histological subtype and treatment modality, Ki67 tumor expression was found to be an OS prognosticator in a cohort of 70 PM patients from two different centers. With this international patient cohort, we were able to validate our previous results regarding the prognostic power of Ki67 expression in PM on the one hand, and reproduce our results regarding the limitation of the prognostic power to only the epithelioid subtype on the other hand [17].

Most importantly, for the first time, Ki67 tumor expression was found to predict treatment benefit, achieved by lung-sparing surgery within multimodality therapy. Accordingly, patients with low Ki67 expression demonstrated an excellent outcome of 48.1 months after lung-sparing surgery within multimodality therapy compared to 24.3 months for patients with high Ki67 expression, underlining the desperate need for predictive biomarkers to distinguish those patients who benefit from multimodality treatment from those who do not. These results are re-challenging the findings and conclusion of the MARS 2 trial that showed an OS of 19.3 months in the multimodality group compared to an OS of 24.8 months in the chemotherapy group, and thus, the MARS 2 authors concluded that the indication of surgery should eventually be questioned in PM [20].

According to our recent and previous findings, we hereby suggest that not the surgery but the patient selection for surgery in PM should be questioned. This question should be answered by validating (predictive) biomarkers, and we hope to contribute to this answer with our present manuscript.

Over 10 years ago, our study group was among the first to suggest using predictive biomarkers to select patients most likely to benefit from multimodality therapy. For this purpose, we re-challenged the previously published promising prognostic biomarker Ki67 tumor expression.

According to the new 2021 WHO Classification of Tumors of the Pleura, nuclear grading for epithelioid diffuse mesothelioma has been introduced and it is now recommended to record this and other histologically prognostic features in pathology reports, but due to the fact that this study utilizes retrospective data, we were not able to include and record nuclear grading [27]. In 2024, Galeano et al. demonstrated by digital image analysis that the Ki-67 index was associated with tumor grade as well as mitotic count, stating that the Ki67 prediction of overall survival was comparable to the mitotic score, thus being a potential surrogate for tumor grade [25]. Galeano et al. described that a high Ki-67 index and mitoses were significantly associated with poor OS (*p* = 0.03 and 0.0005, using 30% and 10/2 mm^2^ as cut-offs, respectively) [7,25].

Regarding the cut-offs, we divided the level of Ki67 tumor expression into low (<15%) and high (>15%) to be able to better compare the influence of Ki67 expression to the overall survival and prognosis. The results from our univariate survival analysis, both via log-rank and Cox regression, proved the prognostic power of Ki67. The Ki67 tumor expression yielded significant survival advantages within the group with low Ki67 expression (31.2 vs. 11.1 months, *p* = 0.012; Figure 1b and Table 2), suggesting that indeed high Ki67 expression is associated with a more aggressive biological subtype of malignant disease, resulting in poor patient survival. Ghanim et al. described that patients with a Ki67 expression higher than the median (>15%) had a significantly (*p* < 0.001) shorter median OS (7.5 months) than those with a low Ki67 expression (19.1 months) [17].

The overall survival in our study was 16.1 months. Baud et al. stated a median survival of 12 months for the entire population (95% confidence interval [CI], 10–15) [18], while Belderbos et al. described a median overall survival of 26.5 months [11,28]. To sum up, the prognosis and overall survival in patients with PM is poor, but patients benefit from a multimodality treatment option, as also shown in our Results section.

When comparing the histological subtypes, we could prove a significant impact on survival in patients with the epithelioid histological subtype compared to the non-epithelioid subtype, which is also confirmed in other previous findings [10,11,17,29,30]. Importantly, we hereby describe that low Ki67 expression is only prognostic in the epithelioid but not in the non-epithelioid histological subtype of PM, which is consistent with previous publications [17]. For the non-epithelioid subgrouped patients, we found a median OS of 7.4 vs. 7.8 months, *p* = 0.732, for those with a low and high Ki67 tumor expression, whereas for the epithelioid patients, the median OS was 36.8 vs. 12.3 months with a low and high Ki67 tumor expression.

Additionally, the different therapy options had a significant impact on overall survival. Both multimodality vs. other therapies and in the surgery subgroups yielded strongly significant results (both *p* < 0.001), just as with multimodal therapy vs. others in multivariate analysis (*p* = 0.019). Patients treated with multimodality therapy survived significantly longer than patients treated with a sole treatment option (median OS: 26.1 months vs. 6.7 months, *p* < 0.001). Surgery, in general, was a strong prognosticator. Patients treated with P/D had an overall survival of 25.6 months, whereas those who underwent tumor debulking had an overall survival of 18.1 months.

Indeed, patients who did not undergo surgery as the therapy option had the poorest overall survival of 3.8 months in our patient cohort. Patients who solely received surgery had a median OS of 7.4 months, which is poorer than the overall survival of patients who received C/R/I (OS: 9.2 months). We additionally analyzed the epithelioid PM patients, who were treated surgically, compared to the epithelioid PM patients, who did not receive surgical treatment. In surgically treated epithelioid PM patients, the Ki67 expression was prognostic in median OS (low Ki67: 48 months vs. high Ki67: 17 months, *p* = 0.003), while in not-surgically treated epithelioid PM patients, the Ki67 index was not prognostic in median OS (low Ki67: 1 month vs. high Ki67: 7 months, *p* = 0.379).

Our findings controvert the only recently published results of the Mesothelioma and Radical Surgery 2 (MARS 2) trial, which described sole systemic chemotherapy as being more beneficial regarding adverse events, quality of life and risk of death, even if the OS was similar compared to systemic chemotherapy + surgery (EPD). However, in the MARS 2 trial, patients were just operated extensively (EPD) [20], and therefore, any comparison to our study regarding surgery as a prognostic variable is adulterated.

Furthermore, Ghanim et al. state that after multivariate survival analyses, Ki67 proved to be—besides histology and treatment—an independent prognostic marker in PM (HR: 2.1; *p* < 0.001) [17]. We could not reproduce the independent prognostic power of Ki67 in our study cohort, which is most likely due to the lower number of included patients when compared to our previous study.

However, after multivariate analysis, only the treatment modality remained as an independent prognostic parameter. We showed a strong statistical power whether patients received multimodality treatment or other therapy concepts (HR 0.31, 95% CI 0.12–0.82, *p* = 0.019). In further testing, a significant interaction between Ki67 tumor expression (cut-off 15%) and multimodality treatment vs. other therapy options (interaction term, *p* = 0.031) was found, indicating a predictive relationship between Ki67 expression and multimodality treatment. The overall survival in this treatment subgroup differed strongly: Patients with a low Ki67 expression showed a significantly better survival with median OS of 48.1 months vs. 24.3 months for patients with a high Ki67 expression. In contrast, patients treated with other therapy options and low Ki67 expression showed worse survival with 1.4 month vs. 7.2 months in the subgroup with high Ki67 expression.

In summary, the current study demonstrates that the Ki67 index is an accurate prognostic and, most importantly, predictive marker in epithelioid MPM, indicating the proportion of proliferating tumor cells and reflecting a more aggressive biological behavior translating to a lower treatment responsiveness to multimodality therapy strategies in the clinical setting. Our retrospective study encloses to the increasing amount of data concerning prognostic biomarkers and parameters in managing PM. Considering the need for prognostic and predictive biomarkers to optimize the treatment decision and management of patients with PM, Ki67 tumor expression might facilitate the patient selection for surgical therapy in a multimodal setting and therefore increase prognosis and quality of life of PM patients.

## 5. Conclusions

This multicenter study demonstrates that Ki67 is a reproducible prognostic and predictive factor in epithelioid PM. Therefore, high Ki67 expression as a value for tumor proliferation, and therewith tumor aggressiveness, affects general prognosis negatively in PM patients and, more specifically, patient outcomes after cytoreductive lung-sparing surgery within multimodal treatment. Ki67 seems a promising biomarker for future patient selection for multimodality treatment of PM patients. The determination of high Ki67 expression combined with specific clinical parameters could help to filter out those PM patients who are less likely to benefit from surgery within multimodality therapy.

## 6. Study Limitations

We want to mention our study limitations. Firstly, the therapeutic modalities in a rare disease like pleural mesothelioma have changed and improved in recent years, but we did not relate the different changing therapy options to the time point of PM patient selection. We know this is a disadvantage and can be an analysis bias.

Additionally, according to the new 2021 WHO Classification of Tumors of the Pleura, nuclear grading for epithelioid diffuse mesothelioma has been introduced and it is now recommended to record this and other histologically prognostic features in pathology reports, but due to the fact that this study utilizes retrospective data, we were not able to include and record nuclear grading.

Furthermore, immunotherapy is a relatively new treatment option for patients with PM which was not available in 2008, when we started our analysis of PM patients; therefore, a relevant comparison between different drugs and the correlation with Ki67 and its survival is not possible for our data, but it would be interesting for following studies.

According to our Ki67 expression analysis, we have mixed data, which means we analyzed Ki67 expression in some PM patients before and, in some PM patients, after medical treatment in the multimodality treatment setting. The correspondence between the Ki67 values before and after medical treatment was not analyzed, but it would be meaningful in future studies and investigations.

## Figures and Tables

**Table 1:** Crosstabulation of patient characteristics according to Ki67 expression.

Variable	Subgroup	Ki67 Expression Low (≤15%)	Ki67 Expression High (>15%)	*n* (%)	*p* *
Sex	male	26 (49.1%)	27 (50.9%)	53 (75.7%)	0.571
	female	7 (41.2%)	10 (58.8%)	17 (24.3%)	
Age at diagnosis	≤66 years	17 (48.6%)	18 (51.4%)	35 (50%)	0.811
	>66 years	16 (45.7%)	19 (54.3%)	35 (50%)	
Histological subtype	epithelioid	30 (50%)	30 (50%)	60 (85.7%)	0.241
	non-epithelioid	3 (30%)	7 (70%)	10 (14.3%)	
Staging °	early (I, II)	22 (51.2%)	21 (48.8%)	43 (62.3%)	0.305
	late (III, IV)	10 (38.5%)	16 (61.5%)	26 (37.7%)	
Multimodality treatment vs. others	multimodality	24 (55.8%)	19 (44.2%)	43 (61.4%)	0.067
	others	9 (33.3%)	18 (66.7%)	27 (38.6%)	
Surgery subgroups	none	8 (34.8%)	15 (65.2%)	23 (32.9%)	0.329
	tumor debulking	8 (57.1%)	6 (42.9%)	14 (20%)	
	P/D	17 (51.5%)	16 (48.5%)	33 (47.1%)	

* *p* = two-sided χ^2^ test; ° = missing case (*n* = 1); P/D = pleurectomy/decortication.

**Table 2:** Patient characteristics and their corresponding median overall survival.

	Cut-Off/Subgroup	*n* (%)	Median OS	95% CI	*p* *
Age	≤66 years	35 (50%)	26.6	18.3–34.9	<0.001
	>66 years	35 (50%)	7.3	6.2–8.5	
Sex	male	53 (75.7%)	17.5	1.0–34.0	0.643
	female	17 (24.3%)	12.3	2.8–21.8	
Histology	epithelioid	60 (85.7%)	18.1	3.7–32.5	0.017
	non-epithelioid	10 (14.3%)	7.4	4.1–10.7	
Epithelioid histology	low Ki67	30 (50%)	36.8	15.3–58.2	0.012
	high Ki67	30 (50%)	12.3	8.9–15.7	
Non-epithelioid histology	low Ki67	3 (30%)	7.4	4.6–10.2	0.732
	high Ki67	7 (70%)	7.8	0.0–18.3	
Stage °	early	43 (62.3%)	24.9	15.9–33.9	0.033
	late	26 (37.7%)	9.2	4.4–14.0	
Therapy	multimodal	43 (61.4%)	26.1	18.8–33.5	<0.001
	Sole surgery	4 (5.7%)	7.4	2.9–11.9	
	C/R/I	7 (10%)	9.2	4.1–14.4	
	BSC	16 (22.9%)	3.2	1.8–4.5	
Multimodal vs. others	multimodal	43 (61.4%)	26.1	18.8–33.5	<0.001
	others	27 (38.6%)	6.7	1.9–11.5	
Surgery subgroups	P/D	33 (70.2%)	25.6	22.3–29.0	<0.001
	tumor debulking	14 (29.8%)	18.1	7.4–28.7	
Ki67 expression	≤15% = low	33 (47.1%)	31.2	6.5–55.8	0.012
	>15% = high	37 (52.9%)	11.1	7.7–14.6	

* *p* = log-rank; ° missing case (*n* = 1); OS = overall survival; C/R/I = chemo-, radio- or immunotherapy; BSC = best supportive care; P/D = pleurectomy/decortication.

**Table 3:** A multivariate (Cox regression) model consisting of age, sex, stage, multimodal vs. others, and Ki67 (*n* = 60).

Variable	Cut-Off/Subgroups	HR	95% CI	*p* *
Age	metric	1.02	0.98–1.05	0.343
Sex	female, male	0.95	0.45–2.02	0.902
Stage	early, late	1.18	0.56–2.50	0.669
Multimodal vs. others	multimodal, others	0.31	0.12–0.82	0.019
Ki67	15%	1.62	0.77–3.40	0.205

HR = Hazard ratio; CI = confidence interval; *p* * = multivariate Cox regression.

## Data Availability

Upon reasonable request, all data and material are available from the corresponding authors.
